# Immunogenicity and safety of a booster dose of a quadrivalent meningococcal tetanus toxoid-conjugate vaccine (MenACYW-TT) in adolescents and adults: a Phase III randomized study

**DOI:** 10.1080/21645515.2020.1733867

**Published:** 2020-03-25

**Authors:** Germán Áñez, James Hedrick, Michael W. Simon, Shane Christensen, Robert Jeanfreau, Eddy Yau, Judy Pan, Emilia Jordanov, Mandeep S. Dhingra

**Affiliations:** a Global Clinical Sciences, Sanofi Pasteur, Swiftwater, PA, USA; b Kentucky Pediatric/Adult Research, Bardstown, KY, USA; c Private Practice, Nicholasville, KY, USA; d J. Lewis Research, Salt Lake City, UT, USA; e MedPharmics, Metairie, LA, USA; f Global Biostatistical Sciences, Sanofi Pasteur, Toronto, ON, Canada; g Global Biostatistical Sciences, Sanofi Pasteur, Swiftwater, PA, USA

**Keywords:** *Neisseria meningitidis*, meningococcus, meningococcal quadrivalent conjugate vaccine, vaccination, booster dose, adolescents, adults, immunization, invasive meningococcal disease

## Abstract

The quadrivalent meningococcal tetanus toxoid-conjugate vaccine (MenACYW-TT) was assessed as a booster in this Phase III trial (NCT02752906). Quadrivalent meningococcal conjugate vaccine (MCV4)-primed individuals aged ≥15 y (n = 810) were randomized 1:1 to receive a single booster dose of MenACYW-TT (n = 403) or a licensed MCV4 (Menactra®; MCV4-DT [n = 407]). Serum bactericidal antibody assay with human complement (hSBA) was used to measure functional antibodies against serogroups A, C, W, and Y at baseline and Day 30 post-vaccination. Proportions of participants achieving seroresponse (post-vaccination titer ≥1:16 for those with baseline titer <1:8 or ≥4-fold increase in post-vaccination titer for those with baseline titer ≥1:8) were determined. Safety data were collected for 180 d post-vaccination. Non-inferiority of the immune response was demonstrated for MenACYW-TT compared with MCV4-DT based on the proportion of participants achieving hSBA vaccine seroresponse for each of the meningococcal serogroups at Day 30. Moreover, ≥99% of participants in both study groups had hSBA titers ≥1:8 for the four meningococcal serogroups at Day 30. Reactogenicity profiles were comparable between groups. These Phase III data in adolescents and adults show that MenACYW-TT boosts the immune response in those primed with MCV4 vaccines 4–10 y previously, irrespective of whether MCV4-DT or MCV4-CRM was used for priming.

## Introduction

*Neisseria meningitidis*, a Gram-negative diplococcus, is exclusively pathogenic for humans. The course of infection with *N. meningitidis* is often rapid, where patients can develop invasive meningococcal disease (IMD), and succumb to the infection in just a few hours. Further, survivors frequently suffer sequelae that can range from limb necrosis requiring amputation to cognitive impairment.^1-3^ There are 12 serogroups of *N. meningitidis*, classified according to their capsular polysaccharide composition. Serogroups A, B, C, W, X, and Y are the most prevalent cause of IMD worldwide; the geographical distribution of the serogroups is dynamic and varies by region.^4^ The epidemiological pattern of IMD necessitates the use of multivalent meningococcal vaccines that provide protection against a wide range of disease-causing serogroups.

Quadrivalent (serogroups A, C, W, and Y) conjugate meningococcal vaccines (MCV4) are used worldwide for the prevention of IMD caused by meningococcal serogroups A, C, W, and Y. In the USA, an MCV4 conjugated to diphtheria toxoid, MCV4-DT (Menactra®, Sanofi Pasteur; not licensed in Europe) was first licensed in 2005; since then MCV4 has been recommended for routine use by the Advisory Committee on Immunization Practices (ACIP) of the Centers for Disease Control and Prevention (CDC) for prevention of IMD in adolescents (11–18 y). Another MCV4, conjugated to the diphtheria protein CRM_197_, MCV4-CRM (Menveo®, GlaxoSmithKline), was licensed in the USA and in Europe in 2010. In October 2010, the ACIP updated recommendations to include a booster MCV4 dose at age 16 y for adolescents previously vaccinated at age 11–12 y.^5^ For adolescents vaccinated at age 13–15 y, administration of a one-time booster dose is recommended, preferably at age 16–18 y.^6^ Several European countries also include recommendations for MCV4 vaccination in meningococcal vaccine-naïve adolescents, or in those primed with monovalent serogroup C vaccines or MCV4s.^7^ Given the geographical variability in meningococcal infection, booster vaccinations are recommended for those with increased susceptibility to infection.^6,8,9^ Older age groups, those who are immunocompromised, or travelers such as Hajj pilgrims (for whom MCV4 vaccination and booster dose after 3 y of first dose is mandatory) have all been found to benefit from a booster vaccination with an MCV4 vaccine to prevent a potential decrease in vaccine efficacy.^10-12^

MenACYW-TT is an investigational quadrivalent meningococcal vaccine conjugated to tetanus toxoid intended for use in all individuals aged ≥6 weeks. The aim of this Phase III study was to compare the immunogenicity and safety of a booster dose of MenACYW-TT with that of the licensed MCV4-DT in adolescents and adults (aged ≥15 y) primed with an MCV4 in the previous 4–10 y.

## Methods

### Study design and participants

This was a Phase III, modified double-blind, randomized, parallel-group, active-controlled, multicenter trial designed to compare the immunogenicity and safety of a booster dose of MenACYW-TT with that of a booster dose of MCV4-DT in MCV4-primed adolescents and adults (NCT02752906). The study was conducted between 15 April 2016 and 2 December 2016 at 29 sites in the USA and one in Puerto Rico.

Healthy adolescents and adults aged ≥15 y who had documented evidence of receiving one dose of an MCV4 vaccine (MCV4-DT or MCV4-CRM) at age 10 y or older, 4–10 y previously, were eligible for inclusion. Major exclusion criteria included participants that: had received previous booster vaccination with either an investigational or approved meningococcal vaccine; planned to participate in another clinical trial or had received any vaccine in the 4 weeks preceding the trial vaccination; had a history of meningococcal infection; were at high risk for meningococcal infection during the trial; had known systemic hypersensitivity to any of the vaccine components, or history of a life-threatening reaction to the vaccine(s) used in the trial or to a vaccine containing any of the same substances; or had a personal history of Guillain-Barré syndrome, or of an Arthus-like reaction after vaccination with a tetanus toxoid-containing vaccine. All participants (or their parent or guardian) signed an informed consent form and in the case of participants aged <18 y, a signed assent form was also required.

Participants were randomized in a 1:1 ratio through an interactive voice response system to receive a single booster dose of MenACYW-TT or MCV4-DT, administered on Day 0. The vaccine administrator was un-blinded to the vaccine, while the participant, the rest of the study personnel, and laboratory technicians processing the samples remained blinded to the intervention.

All participants provided blood samples at baseline (pre-booster vaccination; Day 0) and at Day 30 (+14 d) post-booster vaccination for immunogenicity assessment by serum bactericidal antibody assay using human complement (hSBA). The first 120 participants enrolled and randomized formed a subset from which an additional blood sample was obtained on Day 6 (±1 day) post-vaccination. A subset of 200 participants out of the entire study population also had their baseline and Day 30 samples evaluated by serum bactericidal antibody assay using baby rabbit complement (rSBA).

The conduct of this trial was compliant with the standards established by the Declaration of Helsinki and the International Conference on Harmonization (ICH) guidelines for good clinical practice (GCP) as well as with all local and/or national regulations and directives. The study protocol and subsequent amendments were reviewed and approved by Independent Ethics Committees or Institutional Review Boards at each study site.

Each 0.5 mL intramuscular dose of MenACYW-TT (Sanofi Pasteur) contained 10 µg of meningococcal capsular polysaccharides from each serogroup (A, C, Y, and W) conjugated to approximately 55 µg of tetanus toxoid protein carrier. Each 0.5 mL intramuscular dose of MCV4-DT (Menactra®, Sanofi Pasteur) contained 4 µg of meningococcal capsular polysaccharides from each serogroup (A, C, W, and Y) conjugated to approximately 48 µg of diphtheria toxoid protein carrier. Both vaccines were supplied as a liquid and unadjuvanted and both vaccinations were administered to the deltoid muscle.

### Immunogenicity

The primary objective of this trial was to demonstrate the non-inferiority of the MenACYW-TT booster vaccine seroresponse to meningococcal serogroups A, C, W, and Y measured by hSBA at Day 30 in MCV4-primed participants, compared with MCV4-DT booster. The hSBA (Global Clinical Immunology; Sanofi Pasteur; Swiftwater, PA, USA) and rSBA (Public Health England, Manchester, UK) assays were conducted on sera, with serogroup-specific meningococcal bacteria and human or rabbit complement as described in detail elsewhere.^13,14^ The lower limit of quantitation of both assays was a titer of 1:4. hSBA seroresponse was defined as post-vaccination titers ≥1:16 for those with baseline titer <1:8 or ≥4-fold increase in post-vaccination titer for those with baseline titer ≥1:8.

Secondary and observational objectives included the evaluation of hSBA vaccine seroresponse to all four vaccine serogroups in serum collected at Day 6 after vaccination in a subset of 120 participants, the evaluation of the antibody titers (seroprotection levels and geometric mean titers [GMTs]) to each serogroup measured using hSBA on Day 0 and Day 30 post-booster vaccination and antibody titers at Day 0 and Day 30 after vaccination measured by rSBA in a subset of 200 participants. For hSBA, antibody titers ≥1:8 were considered seroprotective for each serogroup; although a titer of ≥1:4 could be considered protective, the higher titer was chosen as a conservative assumption.^15^ Participants were considered as seroprotected with post-vaccination rSBA titers ≥1:8, which correlates with protection against IMD.^16^ Seroresponse with the rSBA was defined as a post-vaccination titer ≥1:32 for participants with pre-vaccination rSBA titers <1:8 or ≥4-fold increase in post-vaccination titer for those with baseline titer ≥1:8. Both hSBA and rSBA assays were used to assess functional antibody levels due to their acceptability by different regulatory bodies across the world, and to provide global relevance for the results even though the study was conducted in the USA.

Additional planned analyses included the assessment of hSBA seroresponses in each vaccine group by age group at booster vaccination (≥15 to <18 y or ≥18 y), by time elapsed since priming vaccination (4 to <7 y or 7 to 10 y), and by type of MCV4 received at priming (MCV4-DT or MCV4-CRM).

### Safety

Solicited adverse event (AE) information was collected for 7 d after vaccination in diary cards. Unsolicited AE information was collected from Day 0 to Day 30 (+14 d). Solicited AEs were prelisted in the case report form (CRF), reported post-vaccination, and considered to be vaccine-related. Serious AE (SAE) information was collected from Day 0 through to Day 180 after vaccination.

### Statistical analyses

A total of 800 participants were planned to be enrolled in two study groups. With an estimated 15% attrition rate, the study would have had 340 evaluable participants in both study groups. The study was planned to have more than 99.9% power to declare the non-inferiority of the MenACYW-TT seroresponse to that of MCV4-DT based on hSBA antibody titers elicited against each serogroup.

All immunogenicity analyses were performed on the per-protocol analysis set (PPAS), which is a subset of the full analysis set with no major protocol deviations. The vaccine seroresponses to each of the serogroups A, C, W, and Y were tested separately. If the lower limit of the two-sided 95% confidence interval (CI) of the difference in vaccine seroresponse for a given serogroup between the two study groups was more than –10%, then the inferiority assumption for that serogroup was rejected.

Categorical variables were summarized and presented by frequency counts, percentages, and CIs. The 95% CIs of point estimates were calculated using the normal approximation for quantitative data and the exact binomial distribution (Clopper–Pearson method) for percentages.

All safety analyses were performed on the safety analysis set, which included all participants who had received at least one vaccine dose and had safety data available. The main parameters for the safety endpoints were described by frequency counts, percentages, and 95% CIs.

## Results

### Study participants

**Figure 1.**
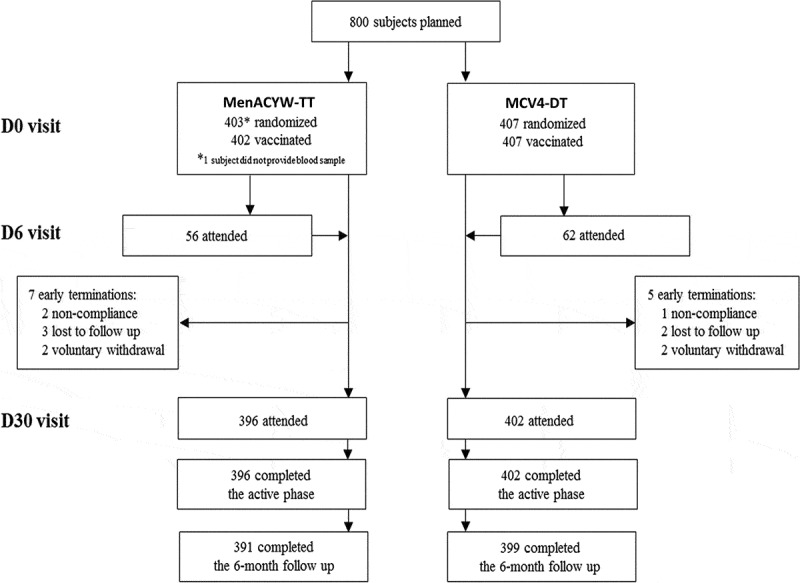
Study flow diagram. D, Day.

A total of 810 participants were enrolled and randomly allocated to the vaccine groups (MenACYW-TT, n = 403; MCV4-DT, n = 407). Of these, 809 (99.9%) participants received their allocated vaccine and 798 (98.5%) participants provided a blood sample at the Day 30 visit and completed the trial. There were no early terminations due to an AE or SAE (Figure 1). A total of 790 (97.5%) participants attended the Day 180 safety follow-up (Figure 1). The demographics of the randomized participants were similar between the vaccine groups (Table 1).

**Table 1.** Participant demographics (all randomized participants).

	MenACYW-TT (N = 403)	MCV4-DT (N = 407)	All (N = 810)
Sex: n (%)			
Male	196 (48.6)	207 (50.9)	403 (49.8)
Female	207 (51.4)	200 (49.1)	407 (50.2)
Sex ratio: Male/Female	0.95	1.04	0.99
Age (y)			
Mean (SD)	20.0 (5.96)	19.9 (5.59)	20.0 (5.77)
Min, Max	15.1, 55.5	15.0, 58.7	15.0, 58.7
Racial origin: n (%)			
White	342 (84.9)	340 (83.5)	682 (84.2)
Asian	11 (2.7)	3 (0.7)	14 (1.7)
Black	40 (9.9)	46 (11.3)	86 (10.6)
American Indian or Alaska Native	1 (0.2)	0 (0.0)	1 (0.1)
Native Hawaiian or other Pacific Islander	0 (0.0)	1 (0.2)	1 (0.1)
Mixed origin	8 (2.0)	17 (4.2)	25 (3.1)
Ethnic origin: n (%)			
Hispanic or Latino	63 (15.6)	71 (17.4)	134 (16.5)
Not Hispanic or Latino	339 (84.1)	336 (82.6)	675 (83.3)
Missing	1 (0.2)	0 (0.0)	1 (0.1)

SD, standard deviation.

### Immunogenicity

**Figure 2.**
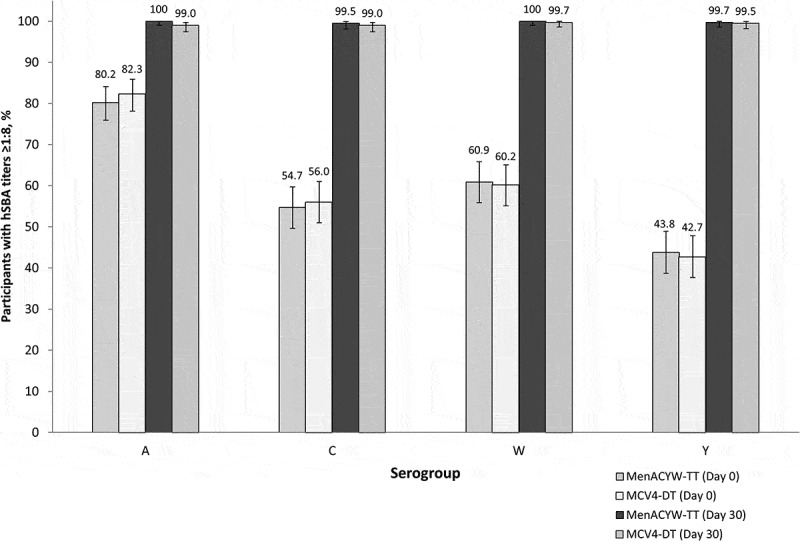
Proportion of participants with hSBA titers ≥1:8 (seroprotection) at Day 0 and Day 30. hSBA, human complement serum bactericidal antibody assay error bars indicate 95% CI.

Non-inferiority of the immune response was demonstrated for the MenACYW-TT conjugate vaccine booster compared with the MCV4-DT booster based on the proportion of participants achieving an hSBA vaccine seroresponse for serogroups A, C, W, and Y at Day 30 post-booster vaccination (Table 2). Baseline hSBA titers were comparable between groups. At Day 30, hSBA GMTs for all serogroups were higher than those at baseline in both groups, and the hSBA GMTs after MenACYW-TT booster were higher than those after MCV4-DT booster for all serogroups (Table 3). At Day 30 the proportion of participants with hSBA titers ≥1:8 (seroprotection) increased from baseline for all serogroups and in both vaccine groups; ≥99% of participants from both groups had seroprotective titers 30 d post-booster vaccination (Figure 2).

**Table 2.** Proportion of participants achieving hSBA vaccine seroresponse^a^ at Day 30.

	MenACYW-TT (N = 384)			MCV4-DT (N = 389)			MenACYW-TT – MCV4-DT		
Serogroup	n/M	%	(95% CI)	n/M	%	(95% CI)	Difference, %	2-sided 95% CI for Difference	Non-inferior^b^
A	354/384	92.2	(89.0, 94.7)	339/389	87.1	(83.4, 90.3)	5.0	(0.735, 9.38)	Yes
C	373/384	97.1	(94.9, 98.6)	357/389	91.8	(88.6, 94.3)	5.4	(2.16, 8.76)	Yes
W	377/384	98.2	(96.3, 99.3)	353/389	90.7	(87.4, 93.4)	7.4	(4.30, 10.9)	Yes
Y	374/384	97.4	(95.3, 98.7)	372/389	95.6	(93.1, 97.4)	1.8	(–0.907, 4.55)	Yes

CI, confidence interval; hSBA, human complement serum bactericidal antibody assay; n, number of participants with titers that meet the hSBA vaccine seroresponse criteria; M, number of participants with valid serology results for the particular serogroup.

^a^Vaccine seroresponse: titer <1:8 at baseline with post-vaccination titer ≥1:16 or titer ≥1:8 at baseline with a ≥ 4-fold increase at post-vaccination; ^b^If the lower limit of the two-sided 95% CI of the difference was more than –10% for each serogroup, the inferiority hypothesis was rejected.

**Table 3.** Geometric means of hSBA titers at baseline (Day 0) and Day 30.

		MenACYW-TT (N = 384)			MCV4-DT (N = 389)			
Serogroup	Time Point	M	GMT	(95% CI)	M	GMT	(95% CI)	GMTR
A	**Day 0**	384	13.7	(12.2, 15.5)	389	15.1	(13.5, 16.9)	
	**Day 30**	384	497	(436, 568)	389	296	(256, 343)	1.68
C	**Day 0**	384	11.0	(9.32, 13.1)	389	10.6	(9.10, 12.4)	
	**Day 30**	384	2618	(2227, 3078)	389	599	(504, 711)	4.37
W	**Day 0**	384	9.76	(8.46, 11.2)	389	10.6	(9.21, 12.2)	
	**Day 30**	384	1747	(1508, 2025)	389	723	(614, 853)	2.42
Y	**Day 0**	384	7.70	(6.56, 9.04)	389	7.27	(6.21, 8.50)	
	**Day 30**	384	2070	(1807, 2371)	389	811	(699, 941)	2.55

CI, confidence interval; GMT, geometric mean titer; GMTR, geometric mean titer ratio (MenACYW-TT/MCV4-DT); hSBA, human complement serum bactericidal antibody assay; M, number of subjects with valid serology results for the particular serogroup and time point; N, number of participants in the per-protocol analysis set.

To evaluate the rapidity of booster response, samples were tested at Day 6 post-booster vaccination in a subset of participants, the vaccine seroresponse and seroprotection rates at Day 6 following booster vaccination were similar between the MenACYW-TT and MCV4-DT groups (Table 4). The Day 6 hSBA GMTs were also similar between the vaccine groups for the four meningococcal serogroups (Table S1).

**Table 4.** Proportion of patients achieving hSBA vaccine seroresponse^a^ or seroprotection^b^ at Day 6.

	MenACYW-TT (N = 55)			MCV4-DT (N = 62)		
Serogroup	n/M	%	(95% CI)	n/M	%	(95% CI)
Seroresponse^a^						
A	40/55	72.7	(59.0, 83.9)	41/62	66.1	(53.0, 77.7)
C	46/55	83.6	(71.2, 92.2)	54/62	87.1	(76.1, 94.3)
W	52/55	94.5	(84.9, 98.9)	52/62	83.9	(72.3, 92.0)
Y	50/55	90.9	(80.0, 97.0)	52/62	83.9	(72.3, 92.0)
Seroprotection^b^						
A	53/55	96.4	(87.5, 99.6)	60/62	96.8	(88.8, 99.6)
C	53/55	96.4	(87.5, 99.6)	60/62	96.8	(88.8, 99.6)
W	54/55	98.2	(90.3, 100.0)	61/62	98.4	(91.3, 100.0)
Y	54/55	98.2	(90.3, 100.0)	60/62	96.8	(88.8, 99.6)

CI, confidence interval; hSBA, human complement serum bactericidal antibody assay; n, number of participants with titers that meet the hSBA vaccine seroresponse criteria; M, number of participants with valid serology results for the particular serogroup.

^a^Vaccine seroresponse: titer <1:8 at baseline with post-vaccination titer ≥1:16 or titer ≥1:8 at baseline with a ≥ 4-fold increase at post-vaccination; ^b^seroprotection: titers ≥1:8.

hSBA vaccine seroresponse in the MenACYW-TT group was similar regardless of the age at time of booster administration (≥15 to <18 y or ≥18 y; Table S2) or the time elapsed since priming vaccination (4 to <7 y or 7 to 10 y; Table S3), or which MCV4 vaccine was used for priming (MCV4-DT or MCV4-CRM; Table S4); the proportion of participants with hSBA seroresponse was also similar between the vaccine groups when assessed by each of these cutoffs.

In the subset of participants for whom rSBA titers were measured, rSBA GMTs for all serogroups were similar between vaccine groups at baseline, and increased, albeit to higher values in the MenACYW-TT group than the MCV4-DT group, at Day 30 (Table S5). The proportion of participants with rSBA titers ≥1:128 increased from Day 0 to Day 30 for all serogroups in both groups, with ≥98% of participants achieving rSBA titers ≥1:128 across the four serogroups (Table S6).

### Safety

Similar reporting rates were observed in the two groups for each safety endpoint (Table 5). The most common solicited injection site reaction was pain (44.7% [178/398] in the MenACYW-TT group and 48.8% [196/402] in the MCV4-DT group), and the most common solicited systemic reactions were headache (37.9% [151/398] in the MenACYW-TT group and 33.3% [134/402] in the MCV4-DT group), and myalgia (36.7% [146/398] in the MenACYW-TT group and 38.8% [156/402] in the MCV4-DT group), all were Grade 1 or Grade 2 in severity, and transient. There were no discontinuations for AEs. Nine participants reported SAEs during the 6-month follow-up: five participants in the MenACYW-TT group and four participants in the MCV4-DT group. None of the SAEs were considered related to the vaccine, and none led to discontinuation from the trial. No deaths were reported during the study.

**Table 5.** Summary of solicited reactions within 7 d after injection and unsolicited adverse events within 30 d post-booster vaccination.

	MenACYW-TT (N = 402)				MCV4-DT (N = 407)			
Solicited reaction within 7 d post-vaccination:	n/M	%	(95% CI)			n/M	%	(95% CI)
Solicited reaction	256/398	64.3	(59.4, 69.0)			263/402	65.4	(60.5, 70.1)
Grade 3 solicited reaction	20/398	5.0	(3.1, 7.7)			22/402	5.5	(3.5, 8.2)
Solicited injection site reaction	185/398	46.5	(41.5, 51.5)			198/402	49.3	(44.3, 54.3)
Grade 3 injection site reaction	4/398	1.0	(0.3, 2.6)			8/402	2.0	(0.9, 3.9)
Solicited systemic reaction	220/398	55.3	(50.2, 60.2)			218/402	54.2	(49.2, 59.2)
Grade 3 systemic reaction	20/398	5.0	(3.1, 7.7)			20/402	5.0	(3.1, 7.6)
Unsolicited adverse event within 30 d post-vaccination:	n	%	(95% CI)	n AEs	n	%	(95% CI)	n AEs
Immediate unsolicited AE	2	0.5	(0.1, 1.8)	2	0	0.0	(0.0, 0.9)	0
Grade 3 immediate unsolicited non-serious AE	0	0.0	(0.0, 0.9)	0	0	0.0	(0.0, 0.9)	0
Immediate unsolicited AR	2	0.5	(0.1, 1.8)	2	0	0.0	(0.0, 0.9)	0
Grade 3 immediate unsolicited non-serious AR	0	0.0	(0.0, 0.9)	0	0	0.0	(0.0, 0.9)	0
Unsolicited AE	106	26.4	(22.1, 31.0)	165	105	25.8	(21.6, 30.3)	166
Unsolicited AR	12	3.0	(1.6, 5.2)	16	12	2.9	(1.5, 5.1)	14
Unsolicited non-serious AE	105	26.1	(21.9, 30.7)	164	103	25.3	(21.2, 29.8)	164
Grade 3 unsolicited non-serious AE	15	3.7	(2.1, 6.1)	16	18	4.4	(2.6, 6.9)	23
Unsolicited non-serious AR	12	3.0	(1.6, 5.2)	16	12	2.9	(1.5, 5.1)	14
Grade 3 unsolicited non-serious AR	0	0.0	(0.0, 0.9)	0	2	0.5	(0.1, 1.8)	3
Unsolicited non-serious injection site AR	5	1.2	(0.4, 2.9)	5	6	1.5	(0.5, 3.2)	6
Grade 3 unsolicited non-serious injection site AR	0	0.0	(0.0, 0.9)	0	1	0.2	(0.0, 1.4)	1
Unsolicited non-serious systemic AE	103	25.6	(21.4, 30.2)	159	98	24.1	(20.0, 28.5)	158
Grade 3 unsolicited non-serious systemic AE	15	3.7	(2.1, 6.1)	16	17	4.2	(2.5, 6.6)	22
Unsolicited non-serious systemic AR	7	1.7	(0.7, 3.6)	11	6	1.5	(0.5, 3.2)	8
Grade 3 unsolicited non-serious systemic AR	0	0.0	(0.0, 0.9)	0	1	0.2	(0.0, 1.4)	2
SAE	1	0.2	(0.0, 1.4)	1	2	0.5	(0.1, 1.8)	2

AE, adverse event; AR, adverse reaction; n, number of participants experiencing the endpoint; number of participants with available data for the relevant endpoint; N, number of participants in the safety analysis set; SAE, serious adverse event.

Unsolicited AE also includes immediate and serious unsolicited AEs; Unsolicited non-serious AE includes any unsolicited AE that is non-serious.

## Discussion

This Phase III study demonstrated the non-inferiority of the investigational quadrivalent meningococcal tetanus toxoid-conjugate vaccine, MenACYW-TT, versus MCV4-DT as a booster in MCV4-primed adolescents and adults aged ≥15 y in the USA and Puerto Rico. The MenACYW-TT booster dose, administered 4–10 y after a priming MCV4 vaccination at age ≥10 y, was immunogenic and well tolerated, and did not generate any safety concerns or safety signals. Overall, the safety profile of MenACYW-TT was comparable to that of MCV4-DT.

MCV4 vaccines prime the immune system and elicit immunologic memory. However, the presence of detectable circulating antibody appears to be important for protection against *N. meningitidis*. Immunity following vaccination with MCV4 may wane over time; in a case-controlled surveillance study of MCV4-DT recipients in the USA vaccine effectiveness was 79% (95% CI 49–91%) for adolescents vaccinated <1 y before, 69% (95% CI 44, 83%) for adolescents vaccinated 1 to <3 y before, and 61% (95% CI 25, 79%) for adolescents vaccinated 3 to <8 y before.^17^ The waning of hSBA titers has also been demonstrated following vaccination with MCV4-CRM,^18^ which led to the recommendation by ACIP in the US for a booster dose at 16 y.^19^ For the same reason, in countries such as the UK, the Netherlands, and Australia, which have different recommended vaccines and schedules, booster vaccinations have been found to be effective in populations considered to be at higher risk of infection.^6,8-10^ As such, there is a need to evaluate the immune response and safety of booster doses of MCV4 vaccines. One study of MCV4-DT administered as a booster to individuals who had received the same vaccine 4–6 y previously showed that at baseline the proportion of participants with hSBA titers ≥1:8 for serogroups A, C, W, and Y were 64.5%, 44.2%, 68.5%, and 38.7%, respectively, which increased to ≥99% across all four serogroups following booster dose.^20^ A similar pattern was observed in this study.

This study also demonstrated that MenACYW-TT is capable of eliciting a rapid booster response as evidenced by the high rate of participants achieving hSBA seroresponse at Day 6 post-vaccination, which is indicative of immune memory after priming with a conjugate vaccine. A rapid immune response is desirable in the case of outbreak control, for example, targeting college campuses or other groups of at-risk populations who should have received MCV4 vaccines previously. The MenACYW-TT booster was also shown to be immunogenic regardless of age and time since first meningococcal vaccination, and regardless of the type of MVC4 (i.e. MCV4-DT or MCV4-CRM) used for priming.

There were some limitations to this study; participants were only included if their initial vaccine was 4 to 10 y previous, as such these results may not be applicable to those who received a priming meningococcal vaccine outside of this window. This study did not assess the levels of anti-carrier antibodies, tetanus in the case of MenACYW-TT, however, a phase II study of MenACYW-TT has shown an increase in anti-tetanus antibody levels following vaccination.^21^

This study offers key data about the safety and immunogenicity profile of the MenACYW-TT conjugate vaccine when administered as a booster in an MCV4-primed population. The data strongly support MenACYW-TT conjugate vaccine as an effective booster vaccination, regardless of the type of MCV4 administered for priming. The study provides evidence that a booster dose of the MenACYW-TT conjugate vaccine administered within 4–10 y after an initial dose of an MCV4 vaccine can elicit a fast and robust immune response in adolescents and adults.

## Supplementary Material

Supplemental Material

## Data sharing

Qualified researchers may request access to patient-level data and related study documents including the clinical study report, study protocol with any amendments, blank case report form, statistical analysis plan, and dataset specifications. Patient-level data will be anonymized and study documents will be redacted to protect the privacy of trial participants. Further details on Sanofi’s data-sharing criteria, eligible studies, and process for requesting access can be found at: https://www.clinicalstudydatarequest.com
