# Supplementary material for: Immunogenicity and safety of a booster dose of a quadrivalent meningococcal tetanus toxoid-conjugate vaccine (MenACYW-TT) in adolescents and adults: a Phase III randomized study
Source: Hum Vaccin Immunother. 2020 Mar 25;16(6):1292–8. doi: 10.1080/21645515.2020.1733867 (PMC7482862; doi:10.1080/21645515.2020.1733867)
Supplement: Supplemental Material [file KHVI_A_1733867_SM2659.pdf]

Supplemental Materials

MET56 Investigators

Donald Brandon, San Diego, CA, USA; Marilou Cruz, Downey, CA, USA; Matthew Davis, Rochester, NY, USA; Cheryl Duffy, Hermitage, PA, USA; John Ervin, Kansas City, MO, USA; Bradley Fox, Erie, PA, USA; Neil Fraser, Troy, MI, USA; Nicole George, Fairfield, OH, USA; Hoadley Harris, Fargo, ND, USA; Jorge Jaramillo, La Puente, CA, USA; William Johnston, Birmingham, AL, USA; Katie Julien, South Jordan, UT; Joseph Leader, Woburn, MA, USA; Christopher Peltier, Cincinnati, OH, USA; James Peterson, Salt Lake City, UT, USA; William Randall, Dayton, OH; Kevin Rouse, Jonesboro, AR; Stephen Russell, Lincoln, NE, USA; Clifford Seyler, Tullahoma, TN, USA; Julie Shepard, Dayton, OH, USA; Gerald Shockey, Mesa, AZ, USA; Michael Simon, Nicholasville, KY, USA; Craig Spiegel, Bridgeton, MO, USA; Patrick Yassini, San Diego, CA, USA; Jennifer Kay, Council Bluffs, IA, USA; Claude Ashley, Dothan, AL, USA; Carmen Deseda, San Juan, Puerto Rico.

Supplementary Table 1. Geometric means of hSBA titers at baseline and Day 6

		MenACYW-TT (N=55)			MCV4-DT (N=62)		
**Serogroup**	**Time Point**	**M**	**GMT**	**(95% CI)**	**M**	**GMT**	**(95% CI)**
**A**	**Day 0**	55	12.1	(8.8, 16.7)	62	19.6	(14.1, 27.1)
	**Day 6**	55	173	(102, 294)	62	226	(141, 363)
**C**	**Day 0**	55	8.00	(5.6, 11.4)	62	11.4	(8.01, 16.3)
	**Day 6**	55	334	(191, 583)	62	448	(277, 724)
**W**	**Day 0**	55	8.00	(6.1, 10.6)	62	11.8	(8.21, 17.1)
	**Day 6**	55	499	(293, 850)	62	346	(216, 555)
**Y**	**Day 0**	55	6.30	(4.4, 9.1)	62	9.67	(6.5, 14.5)
	**Day 6**	55	302	(176, 516)	62	335	(219, 512)

CI, confidence interval; hSBA, human complement serum bactericidal antibody assay; M, number of participants with valid serology results for the particular serogroup and time point; N, number of participants in the per-protocol analysis set

Supplementary Table 2. Proportion of participants achieving hSBA vaccine seroresponse[a] at Day 30, by age at time of booster dose

	MenACYW-TT (N=384)			MCV4-DT (N=389)		
**Serogroup**	**n/M**	**%**	**(95% CI)**	**n/M**	**%**	**(95% CI)**
**≥15 to <18 years**						
**A**	183/201	91.0	(86.2, 94.6)	181/201	90.0	(85.1, 93.8)
**C**	196/201	97.5	(94.3, 99.2)	190/201	94.5	(90.4, 97.2)
**W**	197/201	98.0	(95.0, 99.5)	188/201	93.5	(89.2, 96.5)
**Y**	198/201	98.5	(95.7, 99.7)	195/201	97.0	(93.6, 98.9)
**≥18 years**						
**A**	171/183	93.4	(88.8, 96.6)	158/188	84.0	(78.0, 89.0)
**C**	177/183	96.7	(93.0, 98.8)	167/188	88.8	(83.4, 93.0)
**W**	180/183	98.4	(95.3, 99.7)	165/188	87.8	(82.2, 92.1)
**Y**	176/183	96.2	(92.3, 98.4)	177/188	94.1	(89.8, 97.0)

CI, confidence interval; hSBA human complement serum bactericidal antibody assay; n, number of subjects with titers that meet the hSBA vaccine seroresponse criteria; M, number of participants with valid serology results for the particular serogroup and time point; N, number of participants in the per-protocol analysis set

[a]Vaccine seroresponse: titer is <1:8 at baseline with post-vaccination titer ≥1:16 or titer is ≥1:8 at baseline with a ≥4-fold increase at post-vaccination

Supplementary Table 3. Proportion of participants achieving hSBA vaccine seroresponse[a] at Day 30, by time elapsed since priming vaccination

	MenACYW-TT (N=384)			MCV4-DT (N=389)		
**Serogroup**	**n/M**	**%**	**(95% CI)**	**n/M**	**%**	**(95% CI)**
**4 to <7 years**						
**A**	254/276	92.0	(88.2, 94.9)	250/281	89.0	(84.7, 92.4)
**C**	269/276	97.5	(94.8, 99.0)	262/281	93.2	(89.6, 95.9)
**W**	272/276	98.6	(96.3, 99.6)	256/281	91.1	(87.1, 94.2)
**Y**	271/276	98.2	(95.8, 99.4)	274/281	97.5	(94.9, 99.0)
**7 to 10 years**						
**A**	100/108	92.6	(85.9, 96.7)	89/108	82.4	(73.9, 89.1)
**C**	104/108	96.3	(90.8, 99.0)	95/108	88.0	(80.3, 93.4)
**W**	105/108	97.2	(92.1, 99.4)	97/108	89.8	(82.5, 94.8)
**Y**	103/108	95.4	(89.5, 98.5)	98/108	90.7	(83.6, 95.5)

CI, confidence interval; hSBA, human complement serum bactericidal antibody assay; n, number of subjects with titers that meet the hSBA vaccine seroresponse criteria; M, number of participants with valid serology results for the particular serogroup and time point; N, number of participants in the per-protocol analysis set

[a]Vaccine seroresponse: titer is <1:8 at baseline with post-vaccination titer ≥1:16 or titer is ≥1:8 at baseline with a ≥4-fold increase at post-vaccination

Supplementary Table 4. Proportion of participants achieving hSBA vaccine seroresponse[a] at Day 30, by type of MCV4 received at priming (MCV4-DT or MCV4-CRM)

	MenACYW-TT (N=384)			MCV4-DT (N=389)		
**Serogroup**	**n/M**	**%**	**(95% CI)**	**n/M**	**%**	**(95% CI)**
**MCV4-DT**						
**A**	303/327	92.7	(89.3, 95.2)	298/340	87.6	(83.7, 91.0)
**C**	317/327	96.9	(94.4, 98.5)	311/340	91.5	(88.0, 94.2)
**W**	322/327	98.5	(96.5, 99.5)	306/340	90.0	(86.3, 93.0)
**Y**	318/327	97.2	(94.8, 98.7)	325/340	95.6	(92.8, 97.5)
**MCV4-CRM**						
**A**	43/48	89.6	(77.3, 96.5)	31/39	79.5	(63.5, 90.7)
**C**	48/48	100.0	(92.6, 100.0)	36/39	92.3	(79.1, 98.4)
**W**	47/48	97.9	(88.9, 99.9)	37/39	94.9	(82.7, 99.4)
**Y**	48/48	100.0	(92.6, 100.0)	37/39	94.9	(82.7, 99.4)

CI, confidence interval; hSBA, human complement serum bactericidal antibody assay; n, number of subjects with titers that meet the hSBA vaccine seroresponse criteria; M, number of participants with valid serology results for the particular serogroup and time point; N, number of participants in the per-protocol analysis set

[a]Vaccine seroresponse: titer is <1:8 at baseline with post-vaccination titer ≥1:16 or titer is ≥1:8 at baseline with a ≥4-fold increase at post-vaccination

Supplementary Table 5. Geometric means of rSBA titers at baseline and Day 30

		MenACYW-TT (N=384)			MCV4-DT (N=389)		
**Serogroup**	**Time Point**	**M**	**GMT**	**(95% CI)**	**M**	**GMT**	**(95% CI)**
**A**	**Day 0**	91	1097	(724, 1662)	100	1144	(812, 1613)
	**Day 30**	91	10859	(8844, 13333)	100	6608	(5410, 8071)
**C**	**Day 0**	91	15.2	(9.38, 24.5)	100	9.13	(5.90, 14.1)
	**Day 30**	91	11898	(9425, 15021)	100	2665	(1934, 3672)
**W**	**Day 0**	91	141	(74.3, 269)	100	145	(85.0, 247)
	**Day 30**	91	21227	(17199, 26200)	100	9410	(7203, 12294)
**Y**	**Day 0**	91	84.2	(45.7, 155)	100	52.7	(29.0, 95.9)
	**Day 30**	91	9468	(7447, 12037)	100	3848	(2778, 5331)

CI, confidence interval; hSBA, human complement serum bactericidal antibody assay; M, number of participants with valid serology results for the particular serogroup and time point; N, number of participants in the per-protocol analysis set

Supplementary Table 6. Participants with rSBA titers ≥1:128 at baseline and Day 30

		MenACYW-TT (N=384)			MCV4-DT (N=389)		
**Serogroup**	**Time Point**	**n/M**	**%**	**(95% CI)**	**n/M**	**%**	**(95% CI)**
**A**	**Day 0**	84/91	92.3	(84.8, 96.9)	95/100	95.0	(88.7, 98.4)
	**Day 30**	91/91	100.0	(96.0, 100.0)	100/100	100.0	(96.4, 100.0)
**C**	**Day 0**	26/91	28.6	(19.6, 39.0)	23/100	23.0	(15.2, 32.5)
	**Day 30**	91/91	100.0	(96.0, 100.0)	98/100	98.0	(93.0, 99.8)
**W**	**Day 0**	61/91	67.0	(56.4, 76.5)	70/100	70.0	(60.0, 78.8)
	**Day 30**	91/91	100.0	(96.0, 100.0)	100/100	100.0	(96.4, 100.0)
**Y**	**Day 0**	55/91	60.4	(49.6, 70.5)	54/100	54.0	(43.7, 64.0)
	**Day 30**	91/91	100.0	(96.0, 100.0)	98/100	98.0	(93.0, 99.8)

CI, confidence interval; n, number of participants experiencing the endpoint; hSBA, human complement serum bactericidal antibody assay; M, number of participants with valid serology results for the particular serogroup and time point; N, number of participants in the per-protocol analysis set
